# Dimensions of Hope as Mediators Between Negative Events and Recovery of Well-Being in Adults and Elderly

**DOI:** 10.3390/healthcare13243259

**Published:** 2025-12-12

**Authors:** Santo Di Nuovo, Caterina Ugolini, Rita Zarbo, Paola Magnano

**Affiliations:** 1 Department of Educational Sciences, University of Catania, 95131 Catania, Italy; s.dinuovo@unict.it; 2 Department FISPPA-Applied Psychology, University of Padua, 35131 Padova, Italy; caterinaug@gmail.com; 3 Department of Human and Social Sciences, Kore University of Enna, 94100 Enna, Italy; paola.magnano@unikore.it

**Keywords:** hope, stressing events, resilience, well-being, elderly

## Abstract

**Highlights:**

**What are the main findings?**

- Specific dimensions of hope mediate the relationship between stress resulting from negative life events and resilience and well-being.
- This mediation involves different hope components for adults and older adults, while spirituality appears to be non-significant in both groups.

**What are the implications of the main findings?**

- Hope can be increased, even in old age, through specific training to promote resil-ience and the recovery of well-being when dealing with stressful events.
- In training to enhance hope, specific aspects of the construct should be addressed to increase their effectiveness.

**Abstract:**

**Background/Objectives:** Hope plays an important role in coping with difficulties and is predictive of resilience, improving the skills necessary to sustain life plans and well-being, and overcoming stressful situations in adulthood and especially in older age adults. We hypothesised that the dimensions of hope, including spirituality, are connected to personal, educational, and contextual conditions, and play a mediating role in fostering resilience and well-being after stressful events; this mediating role might differ in adulthood and among the elderly. **Methods:** The sample consisted of 100 adults without severe pathologies and living in their own homes, aged between 50 and 86 years (M = 66.08; SD = 8.48). They completed an online survey that included the Stress Event scale, the Comprehensive State Hope Scale, the Resilience Scale for Adults, and the Well-being Profile. The data were analysed using SPSS and JAMOVI software, applying the following statistical tests: *t*-test, ANOVA and mediational model. **Results:** Stressful events influence resilience and well-being differently in adulthood and old age, with non-significant differences due to gender and living conditions. Hope mediates between stress resulting from negative life events and resilience and well-being, but this mediation involves different hope components for adults (trust) and older adults (self-realisation and perception of social support in interpersonal relationships). Spirituality is a non-significant mediator in both age stages. **Conclusions:** Appropriate psychological and psychosocial supports are needed to enhance the mediating potential of hope between stressful events and resilience. The results of our study help clarify which components of hope specifically promote resilience in different conditions of normal old age, differentiating them from those more beneficial for adults.

## 1. Introduction

The cognitive aspects of hope motivate one to pursue goals and life paths deemed important, using active strategies to cope with experienced difficulties [1]. Cognitive orientation toward desired goals is embodied in the various thought paths that pursue these objectives. In contrast, “agentic thinking”—which characterises agency, i.e., the perception of being capable of pursuing goals—initiates and sustains the journey toward them. Thinking directed toward the desired end is associated with a positive sense of meaning in life. Two main dimensions of hope are the desire to attain a goal, along with the belief and trust in the possibility of achieving it [2].

Erikson [3] highlighted the developmental nature of the construct of hope, which serves as the framework through which people develop their identity across the different stages of life. Hope is among the positive virtues that accompany a person throughout their lifespan, contributing to healthy development in childhood and to proactive coping with the crises necessary to move from one developmental stage to the next.

Scioli and Biller [4] presented an articulated model of hope, drawing on the psychological constructs of attachment, self-regulation, and active coping with critical life situations (mastery). They hypothesised that hope is articulated in different dimensions (trust, openness, connectedness, mastery), in conjunction with spirituality as an attitude of transcendence of present life.

The social constructivist approach defined hope as a multilevel construct: a future-oriented network of cognitions and emotions grounded in biological, psychological, and social resources. These aspects differentiate hope from general optimism. Several authors have highlighted the substantial distinction between optimism and hope [5,6,7]. The former consists of a more general, nonspecific perception of desirable outcomes, not necessarily based on an assessment of their actual probability of success or the subject’s active involvement. Hope, instead, has more realistic and defined objectives, with a tendency to be active even in the face of possible difficulties. Agency, therefore, represents the main peculiarity of hope, which involves both the expectations of being able to pursue one’s own goal and the awareness of possessing the motivation and skills necessary to achieve it by identifying effective strategies or action plans, according to what respectively represent the two essential components of the construct: “agency” and “pathways” [8].

Positive health [9] encompasses hope for a better future and a sense of determination to achieve the planned goals [10]. For these reasons, hope has been considered a “deterrent to illness” [11] and a way for coping with stress [12]. Hope has been confirmed to be predictive of resilience, playing an important role in the process of adaptation to suffering [13]. It constitutes a key element in coping with difficulties and in developing subsequent skills to overcome them. These skills are necessary not only to structure and maintain a life project but also to overcome stressful situations that might threaten the individual’s lifespan and overall well-being, especially in adulthood and particularly in the elderly.

### 1.1. Hope in the Elderly

Ageing is a phase of life in which many individuals tend to disconnect from their social and professional roles, withdraw from daily activities and social relationships, and are more likely to experience negative cognitions and emotions, including a sense of hopelessness. Hopelessness is negatively associated with various indicators of psychological well-being along the lifespan and has been included among the main indicators of depression and other emotional disorders (for a review, see [14]). Conversely, hope is strongly associated with a range of psychosocial processes and outcomes, including emotional adjustment, positive affect, life satisfaction, sense of purpose, quality of life, and social support. It may constitute a potential protective factor against the possibility of suicide, particularly by mediating the relationship between suicidal ideation and hopelessness. Hope can help address stress and adversity by influencing the identification of multiple coping strategies, allowing individuals to maintain long-term goals while seeing alternatives to hopelessness. This preserves a personal sense of self-efficacy and a recovered existential meaning, projected toward the future and capable of surviving crises, in adulthood and in old age.

Feldman & Snyder [15] demonstrated that hope, as a component common to all theories of meaning about life, attenuates the correlations between meaning and both depression and anxiety. Hope is an essential need for the elderly, providing resources that enable individuals to cope with problems and losses and to overcome frequent negative events in the last stages of life. It is essential for coping with illnesses, transcending the limitations of ageing [16].

In a study conducted on older adults, hope emerged as a predictor of better physical and mental health, unlike optimism, thus suggesting that what matters is not merely holding a general positive expectation, but rather retaining the capacity to maintain goal-directed thinking and the motivation necessary to achieve such goals [17]. Similarly, a strong positive correlation between hope and resilience was observed in a sample of older adults, reflecting their tendency to maintain a positive outlook despite adversity [18]. Instead, loneliness and the loss of social ties, both common in advanced age, confirm the negative impact of diminished social support on the well-being of older adults [19].

The importance of family presence, broader social support, and their interaction with the components of hope has also been explored [20]. Results indicate that, among older adults, perceived social support exerts both a direct influence on loneliness—by reducing it—and an indirect effect mediated through hope and related personal self-evaluations. Specifically, while the pathways thinking component of hope tends to decline with age, the agency component—that is, the motivation to pursue and achieve one’s goals—appears to remain stable and to constitute the primary factor influencing reductions in perceived loneliness. Indeed, although older adults may experience physical limitations due to age-related functional decline, this deterioration does not appear to affect the development and maintenance of adequate motivation, which strong social support can further strengthen. In this regard, hope helps internalise resources from external sources of support and transform them into positive coping strategies and an improved self-concept, thereby reducing feelings of loneliness. In this way, hope becomes a fundamental positive coping strategy for facing and facilitating adaptation to old age. This adaptation presupposes awareness of age-related changes and the consequent regulation of personal expectations, goals, and actions, as well as an acceptance of oneself and others. Achieving such adaptation requires a process of selecting new goals, optimising functions, and compensating for diminished resources, a process that is supported by the dimension of hope, understood as a goal-directed disposition [21]. Therefore, both social support and hope operate as mediators between activities performed and life satisfaction in older adults, promoting the attainment of a “healthy ageing” or “ageing well”, driven by a renewed desire for life [22].

Positive psychological functioning (including hope and optimism) influences health, engages persons in healthier behaviours beyond their clinical suffering, and enhances the efficacy of treatments [23]. Moreover, hope helps care for others with chronic illnesses. The quality of life, physical and mental health, life satisfaction, and the hope of care recipients were found to be positively associated with caregivers’ hope [24].

Long et al. [25], considering that few studies on hope include older adults outside clinical settings, used data from the Health and Retirement Study (HRS), a large and representative sample of adults aged 50 and above, to evaluate whether positive changes in hope are associated with better subsequent health and well-being. The results demonstrated that a greater sense of hope was associated with better physical health and health behaviour outcomes on some indicators, as well as higher psychological and social well-being. Lower levels of hope are observed in late adulthood (from age 65), coinciding with life events such as retirement—often linked to a sense of loss of one’s role and social usefulness—fewer contacts, possible worsening of health, and life changes within families. Social support and the relational dimension, once again, demonstrate an important role, not only in the genesis of hope but also in its maintenance over the years. Early youth experiences also appear to play a decisive and predictive role in the progression of hope levels in adulthood [19].

### 1.2. Hope and Spirituality

Hope has been described as a spiritual way of seeking meaning, also in social and health difficulties [26]. “A dynamic model of health includes the spiritual self and hope and is of interest to understand the relationship between spirituality and hope” [27] (p. 432), remembering that spirituality is often different from religiosity [28,29].

Davis [30] reported a significant correlation between hope and spirituality in a sample of healthy older adults aged between 60 and 89 years, suggesting that these factors jointly reinforce well-being in a health-promoting cycle. Sharif et al. [31] investigated the relationship between hope, spirituality, and life satisfaction in a sample of 1348 older adults, confirming the significant role of spirituality in coping with the daily challenges associated with ageing and, together with hope, in promoting higher levels of health and life satisfaction. Hope helps people cope with difficult life events by drawing on their spiritual or transcendent nature and/or by experiencing interconnectedness with significant others [32]. A pragmatic model of social hope is based on the transcendent possibilities of the emerging future [33].

Summarising, religiosity is positively correlated with hope, which, in turn, is positively associated with life satisfaction [34]. But what kind of spirituality is more helpful in enhancing hope? The sense of transcendence can refer to a relationship with a superior entity and/or to a connection with others, such as family and social groups [27,35]. As Ricoeur [36] (p. 51) stated, the sacred emerges out of connectivity with others, and is a way of being-in-the-world, re-describing reality in relation to something ‘transcendent’, i.e., beyond the present situation. In this sense, spirituality may help individuals cope with the difficulties associated with ageing by emphasising the importance of meaning in life and social relationships [22].

### 1.3. Hope Components as Possible Mediators of Well-Being

Since hope is linked to well-being [1,12], particularly in the elderly [16,17], we can hypothesise that, in this age, hope mediates between coping with subjective distress and the restoration of well-being. This role aligns with studies clarifying how hope can contribute to personal well-being by enabling individuals to maintain a positive outlook when facing stressful or critical life events [18], thereby mitigating their impact, and by mediating the relationship between perceived loneliness and potential social support [20]. These studies, although still limited in number, allow hope to be understood as a dynamic construct-potentially functioning as a mediator—that can be influenced and elicited by various personal and contextual elements deserving further investigation.

In fact, hope can be experienced in different modes and in different contexts [37]; it can be understood as an individual experience or as a context-dependent process [7]. Internal and external sources of hope should be studied jointly. Therefore, it is interesting to examine whether there are differences in the mediation process between external events and coping, based on hope, social variables, and age (specifically, adulthood versus old age).

## 2. Aims of the Study

The study aims to explore the role of hope in promoting resilience and maintaining psychological well-being among adults and elderly individuals across genders and educational backgrounds. Specifically, we hypothesise that the construct of hope, articulated in its different dimensions (including spirituality):

**H1:**  *is related to personal, educational, and contextual conditions.*

**H2:**  *plays a mediating role in fostering resilience after stressful events and recovering subjective well-being in non-pathological ageing.*

**H3:**  *The mediating role differs between adulthood and old age.*

**Figure 1:**
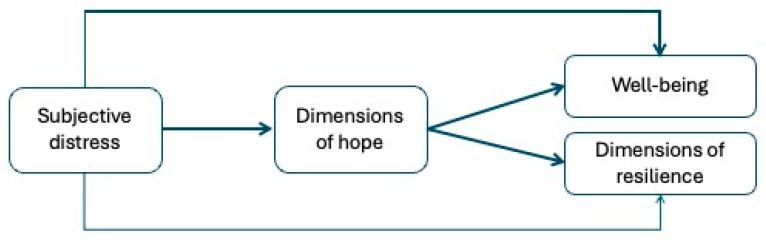
The conceptual model.

Figure 1 presents the conceptual model for H2 and H3 to be tested in two separate groups: adults and the elderly.

## 3. Materials and Methods

### 3.1. Participants and Procedure

The sample size was determined using G*Power software 3.1.9.6 version, with the following parameters: effect size (f^2^) = 0.20; α = 0.05; number of predictors = 1. The required sample size was *n* = 56, which is not far from the groups’ sizes in our study. A total of 100 adults (men = 34; women = 66), aged between 50 and 86 years (M = 66.08; SD = 8.48), were recruited for the study; 52% of the total participants were aged ≤ 65 years (named adults, age range = 50–65, M = 59.7; SD = 4.11) and 48% were aged ≥ 66 years (named elderly, age range = 66–86, M = 73; SD = 6.22). The majority of participants held a university degree (43%) or a high school diploma (33%), while a smaller proportion had completed lower secondary school (9%) or elementary education (15%). Regarding marital status, 65% of participants were married, 15% were separated, 13% were widowed, and 7% were single. Regarding household composition, most participants lived either with their spouse/partner (40%) or with their family, including both their spouse/partner and their offspring (30%). A smaller proportion lived only with their offspring (5%), in their offspring’s home (4%), with a caregiver (2%), or alone (19%). Finally, participants’ employment status was distributed as follows: 54% were employed, 39% were retired, and 7% reported having been engaged exclusively in domestic work throughout their lives. The inclusion criteria were the absence of symptoms of mental deterioration and residence in one’s own home.

Participants were recruited through convenience sampling via multiple dissemination channels, including digital platforms, emails, and elders’ associations. They were fully informed about the study’s aims and methods and were explicitly asked to provide their consent by selecting the option before proceeding with the survey. They completed the online survey on the Google Forms platform between June and October 2025. The study was approved by the Ethics Committee of one of the participating universities.

### 3.2. Instruments

Stressing Events (StEv). This scale has been created ad hoc for the study’s scope, revising Paykel et al.’s [38] Life Events scale, integrating items derived from the indicators used in Long et al.’s [25] study, and the main indicators proposed by DSM as potential causes of traumatic stress disorder. A total of 49 negative events are proposed, categorised into nine areas: work, education, financial or legal issues, mourning, health, logistics, and partner and family relationships. For each event, the recent presence (i.e., in the last 6 months), the less recent presence (in the previous 5 years) has to be marked, and—for the events indicated as present—the perceived severity, on a scale from 1 to 5. The scores of the StEv scale add up to how many recent events (max 49) and how many less recent (max 49) have been marked; how many persistent (both presences marked); and how severe the scored events are perceived (max 245). This last assessment is an index of subjective distress, not an objective one, based on the standard severity of the event (as in Paykel); in this sense, the StEv integrates with the Impact of Event Scale—IES [39]. The reliability of the score of subjective distress was preliminarily assessed, obtaining a good Cronbach’s alpha (α = 0.81) and a Spearman–Brown Coefficient = 0.82.

The Comprehensive State Hope Scale (CHS–S; [28]). The Italian version of the CHS-S [40] is composed of 37 items, for which the respondents receive the following instructions: “My recent thoughts and feelings. This questionnaire deals with your current and recent thoughts and feelings. That is, how you feel today and over the past two weeks”. A 5-point Likert scale indicates levels of hope, ranging from none (0) to extremely strong (4). The dimensions evaluated are spirituality (sample item: “I have felt a spiritual presence”), relationships and social support (sample item: “I can turn to a good friend or family member to help me relax”), trust (sample item: “I feel let down by someone that I trusted”- reversed scoring), Self-realization (sample item: “Everyday I’m getting closer to achieving my dreams”). Cronbach’s alphas and McDonald’s omega of the four dimensions are respectively: spirituality α = 0.94, ω = 0.94; relationships and social support α = 0.86, ω = 0.86; trust α = 0.78, ω = 0.78; self-realisation α = 0.74, ω = 0.75.

Resilience Scale for Adults (RSA [41]; Italian adaptation [42]). The scale is composed of 29 items, measuring six different factors of resilience: personal competence (sample item: “When something unexpected happens … I always find a solution—I always feel disoriented”), social competence (“sample item: I have fun … with other people–by myself”), family cohesion (sample item: “In difficult times my family … looks to the future positively–looks to the future negatively”), social support (sample item: “When I need help … I have no one who can help me—I always have someone who can help me”), future perception (sample item: “My future goals are…clear-unclear) and personal structure (sample item: “When I start a new project … I rarely plan—I prefer to plan.”). The responses are provided on a semantic differential by placing half of the positive and half of the negative semantic differentials to the right. Cronbach’s alphas and McDonald’s omega of the six dimensions are respectively: personal competence α = 0.78, ω = 0.78; social competence α = 0.73, ω = 0.74; family cohesion α = 0.74, ω = 0.77; social support α = 0.72, ω = 0.74; future perception α = 0.73, ω = 0.74; personal structure α = 0.54, ω = 0.57.

Well-being Profile—Short version (WB-Pro; [43]; Italian adaptation [44]). It comprises 15 items with a 9-point Likert scale, from 1 (completely disagree) to 9 (completely agree), that provide a single global index of well-being. Sample item: “I quickly get over and recover from significant life difficulties”. Cronbach’s alphas and McDonald’s omega of the scale are: α = 0.89, ω = 0.90.

### 3.3. Data Analysis

Missing data below 5% [45] were replaced using the series mean method. Means (M), standard deviations (SD) and ranges were then calculated. In addition, Cronbach’s alpha and McDonald’s omega coefficient have been calculated to assess internal consistency of the scale used (cited in the “Instruments” Section); their acceptable thresholds are as follows: a value of less than 0.60 is deemed unacceptable, while a score between 0.60 and 0.70 is considered acceptable. A score above 0.70 indicates good reliability, while a score above 0.80 indicates excellent reliability [46,47].

Student’s *t*-test and one-way ANOVAs were performed to assess differences among subgroups; Spearman’s correlation was utilised to verify the relationships between the selected variables. The normal univariate distribution of the scores was tested by reporting kurtosis and skewness values, along with the Shapiro–Wilk W statistic and its significance. These statistical analyses were performed using SPSS version 25.0 [48]. Then, the mediational analysis was conducted using JAMOVI 2.6.26 [49]. Following the conservative approach suggested by Preacher and Hayes [50] and Fritz and MacKinnon [51], we used bootstrapping with 5000 repetitions to estimate the indirect effects. The authors recommend this method to reduce the risk of Type 1 error and to obtain a more accurate estimate of effects in small samples. The significance of the indirect effects obtained via bootstrapping with 5000 repetitions was reported, along with 95% confidence intervals (CIs).

## 4. Results

### 4.1. Differences by Gender, Age, Instruction and Living Conditions

To verify differences by gender and age, we applied Student’s *t*-test (threshold: *p* < 0.05) to compare the study variables between two groups: men/women (gender differences) and adults/elderly (age differences).

First of all, no gender differences have been found among the study participants (for all the variables *p*_t_ > 0.05); on the contrary, significant differences have been detected between the two age groups (adults and elderly). Cohen’s d [52] is used to express effect size as follows: small (0.2), medium (0.5), and large (0.8). As shown in Table 1, the perception of well-being is significantly lower among the elderly, a trend also observed across almost all dimensions of resilience, specifically future perception, family cohesion and personal competence. While future perception and personal competence are individual resources related to self-achievement, family cohesion is associated with changes in familial relationships across the life, i.e., children leaving the nuclear family. The CHS dimensions do not show any significant differences between the two groups.

The differences in levels of instruction and living conditions were tested using one-way ANOVA (*p* < 0.05). The differences by school level highlight that participants with a higher school level have higher scores in two dimensions of CHS—relationship/social support and self-realisation—as well as in subjective well-being and the structured style of RSA dimension. Furthermore, participants with a higher school level have lower scores in subjective distress. Post-hoc Bonferroni highlighted that these differences are significant, indicating that participants with a lower school level have lower levels in psychosocial resources: hope (relationship/social support and self-realisation), well-being, resilience (personal structure), showing higher levels of subjective distress. In Table 2, we report the comparison results, highlighting the significant differences (in italics). No significant differences were found in living conditions (*p* > 0.05).

### 4.2. Subjective Distress: Distribution and Differences in the Two Age Groups

Across the entire sample, all participants reported experiencing at least one of the 49 assessed events, either in the past six months, over the past five years, or persistently (i.e., in both the past six months and over the past five years). Specifically, 71% of participants reported experiencing at least one event in the past six months, 99% reported at least one event over the past five years, and 37% reported experiencing at least one event persistently.

To examine the overlap among the three temporal exposure types (past six months, past five years, and persistent), the frequencies of all possible combinations of the three exposure types were calculated. The results indicate that 1% of participants experienced events exclusively in the past six months, 23% experienced events exclusively in the past five years, 39% experienced events either exclusively in the past six months or exclusively in the past five years but never persistently, 6% experienced events either exclusively in the past five years or persistently but never exclusively in the past six months, and 31% experienced events across all three temporal categories. Moreover, the results show a higher percentage (with a difference of 7%) of persistent events in the elderly (22%) than in adults (15%). Regarding subjective distress, 23 adults and 27 elderly participants scored above the median (0.23).

Subsequently, we calculated frequencies and percentages separately for the two age groups: adults (aged ≤ 65 years) and the elderly (aged > 66 years). Results indicate a higher prevalence of persistent events among older participants compared to younger participants, a similar prevalence of events over the past five years across both groups, and a higher frequency of recent events (past six months) in the younger group. Specifically, among adults, 73.1% reported at least one event in the past six months, 98.1% reported at least one event over the past five years, and 28.8% reported at least one persistent event. Among the elderly, 68.8% reported at least one event in the past six months, 100% reported at least one event over the past five years, and 45.8% reported at least one persistent event.

To further investigate the overlap between the three temporal exposure types within each age group, the frequencies of all possible combinations were calculated separately.

Among adults, 1.9% of participants experienced events exclusively in the past six months, 23% exclusively in the past five years, 46.2% either exclusively in the past six months or exclusively in the past five years but never persistently, 3.8% either exclusively in the past five years or persistently but never exclusively in the past six months, and 25% experienced events across all three temporal categories. Among the elderly, none of the participants experienced events exclusively in the past six months, 22.9% experienced events exclusively in the past five years, 31.3% either exclusively in the past six months or exclusively in the past five years but never persistently, 8.3% either exclusively in the past five years or persistently but never exclusively in the past six months, and 37.5% experienced events across all three temporal categories.

### 4.3. Correlations

We then verified the zero-order correlations among age, hope, resilience, well-being, and the severity score of the stressful events identified in the study using Spearman’s rho. Table 3 presents the means, standard deviations, skewness, kurtosis, the Shapiro–Wilk test for normality, and correlations between the analysed dimensions.

Most correlations are in the direction expected. Well-being is strongly and positively associated with the main aspects of the CHS dimensions, including trust, relationships, social support, and self-realisation; however, no association has been found with the CHS spirituality dimension. Moreover, well-being is significantly, strongly and positively related to the dimensions of RSA, except for the personal structure, which shows a significant but weaker negative association. Finally, significant and negative associations are observed between (1) some dimensions of hope (trust and self-realisation), well-being with subjective distress and (2) some dimensions of hope (trust and self-realisation), well-being, and some dimensions of resilience (future perception, family cohesion, personal competence) with age.

Subsequently, we decided to separate the two subgroups and test correlations for adults and the elderly, finding the same patterns in both groups.

### 4.4. The Mediational Model

**Figure 2:**
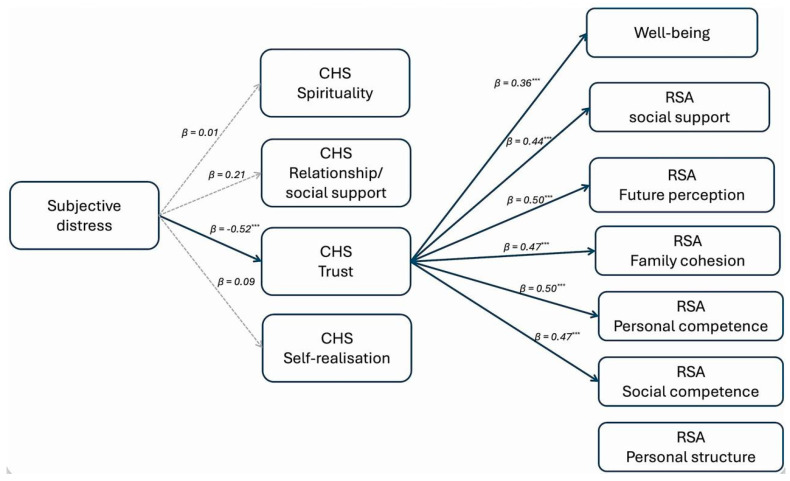
The mediation model tested on adults, *** *p* < 0.001.

**Figure 3:**
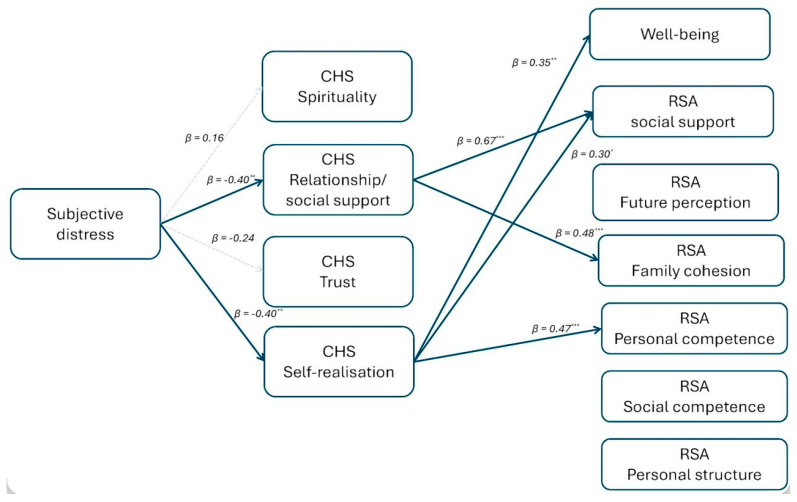
The mediation model tested on the elderly, * *p* < 0.05; ** *p* < 0.01; *** *p* < 0.001.

The testing of the mediational hypothesis was conducted using the bootstrapping method with 5000 repetitions to assess the significance of the indirect effects. We tested the model represented in Figure 1, with subjective distress as a predictor, the dimensions of hope as mediators, and well-being and the dimensions of resilience as outcomes. The model was tested separately on two groups of participants: adults and the elderly, as preliminary variables confirmed statistical differences between these groups for WBP and most RSA variables (i.e., the dependent variables in the model). The statistical models are shown in Figure 2 and Figure 3 (non-significant effects between mediators and outcome variables are omitted for readability).

Among adults, the trust dimension of the CHS fully mediates the relationship between subjective distress and well-being, as well as the following RSA dimensions: social support, future perception, family cohesion, personal competence, and social competence.

In the group of the elderly, the relationship/social support dimension of the CHS is a full mediator of the relationship between subjective distress and social support and family cohesion dimensions of the RSA; the self-realisation dimension of the CHS fully mediates the relationship between subjective distress and well-being and social support and personal competence dimensions of the RSA.

In both groups, the spirituality dimension of the CHS does not act as a mediator, and the personal structure dimension of the RSA is not affected by any of the antecedents. Moreover, no direct effects have been found.

Table 4 and Table 5 report the mediation model’s indirect effects, confidence interval (C.I.) and significance (*p* < 0.05).

## 5. Discussion

The results of our study, in relation to the hypotheses, demonstrate that stressful events impact resilience and well-being differently in adulthood and old age (both without invalidating pathologies), with no significant differences due to gender and living conditions.

As hypothesised based on previous literature [1,4,12,18], hope mediates between stress resulting from negative life events and resilience and well-being. The mechanism underlying the mediating role of hope could be referred to the positive self-evaluations increasing the sense of agency and the life meaning, as suggested by Feldman & Snyder [15]. Moreover, trust in social support appears to reduce loneliness by reinforcing hope [20].

However, this mediation involves different components of hope for adults and older adults. In adults, trust in oneself and in significant others is the component of hope most closely associated with positive influences on resilience and well-being. In non-pathological elderly, the main mediators between stressful events and active coping are self-realisation and the perception of social support in interpersonal relationships [20,22].

Spirituality is not a significant mediator in either group. This is an unexpected outcome, as many studies have confirmed that spirituality helps cope with life stress [30,31,34], including in ageing. We anticipated that possessing a self-transcendence dimension would act as a protective factor against crises, also aligning with the theory of gerotranscendence [53,54]. In fact, gerotranscendence–taking up Erikson’s [3] suggestions–is conceptualized as a shift in meta-perspective from a material and rational vision to a more transcendent worldview and sense of cosmic connection; this shift could involve a redefinition of one’s perception of time and space, and a conscious movement towards disengagement from unnecessary social interactions, self-centredness, and material possessions [55].

However, in recent times, people in active ageing have become less motivated to cut bonds with society and turn inward, as suggested by Tornstam [53]. The process of secularisation could have modified the meaning of transcendence, also restructuring its relationship with the constructs of religiosity and spirituality [56]. This could explain why empirical research on the psychological well-being of people who identify with spirituality, and in particular those who self-identify as more spiritual than religious, has yielded inconclusive findings”, some studies even reporting negative effects. Considering the specific meaning of spirituality assessed by the Scioli’s test used in the research, we can hypothesize that it reflects a “vertical” dimension of contact with a higher entity, which in Western culture more “secular” aspects of hope are affirming. This interpretation is supported by the significance as mediators of the “immanent” dimensions of hope as transcendent possibilities of the emerging future [33]: trust in oneself and others in adults, self-realization and social support in the elderly; dimensions included in the aforementioned definition of spirituality by Ricoeur [36] and Oliver et al. [22]. This kind of spirituality can be categorised as “belief and belonging” [57], and as a connection with other persons, the inner self, and an ethical orientation toward humanity and nature [58].

## 6. Limitations

Some limitations must be acknowledged in our study. Convenience sampling limits the generalisability of the results; furthermore, the use of self-report measures can be vulnerable to desirability bias. Additionally, only adults and elderly individuals without serious chronic illnesses or recent hospitalisation were included; in such cases, the mediating role of the factors constituting hope might differ. To explore potential differences, we plan to replicate the model with a sample of institutionalised elderly people. Another limitation arises from the choice of the spirituality construct assessed in the test we employed. Different constructs may be characteristic of other cultural contexts, such as Anglo-Saxon and Eastern cultures, as noted by Wixwat and Saucier [59]. Spirituality has multiple meanings, as it is a highly heterogeneous construct.

## 7. Conclusions

The results of our study, highlighting aspects of the mediating role that hope can play in responding to critical events to maintain or restore well-being, have implications for both stress coping theory and the practical applications of psychological interventions.

According to Wienand et al. [60] (p. 184), “hope is not a mere coping strategy, but also a positive commitment towards life, a disposition which can accommodate fear, anxiety, and lucidity, and is sometimes related with confidence in or reliance on transcendence”. Since hope is a predictor of well-being and is linked to personality [61], it can be promoted and enhanced throughout the lifespan to prevent physical and psychological diseases and foster well-being, even in the later stages of life. Appropriate psychological and psychosocial supports are needed to boost the mediating potential of hope between stressful events and resilience. In particular, considering that the future orientation of hope appears to be the key component enabling older adults to overcome the sense of limitation and the physical constraints imposed by ageing, psychological interventions can be designed to target this dimension, focusing on reawakening in clients the hope of overcoming present difficulties and pursuing new, recalibrated goals [62].

Hernandez and Overholser [63] conducted a review of the main interventions targeting hope in older adults. Among these, the following can be highlighted: group-based Cognitive Behavioural Therapy sessions—based on changing unhelpful beliefs and learning new coping skills—which are effective in reducing experiences of hopelessness and associated depressive or suicidal thoughts; supportive intervention, including psychoeducational strategies based on sharing information and experiences; exercises interventions; combination treatments, involving the simultaneous use of both pharmacologic medications and psychological intervention; and life review-based interventions.

Included in this latter category are therapies widely supported in the scientific literature, such as Dignity Therapy [64], originally developed within the context of palliative care but also applied beyond it, for instance, in work with older adult populations. This intervention focuses on fostering a sense of generativity regarding one’s past life, its meaning, and legacy, to strengthen hope even in the final part of one’s life. It involves conducting life story interviews to develop a generativity document that the patient can choose to share with others [63].

A particularly well-known therapeutic approach is Snyder’s Hope Therapy. This intervention consists of group sessions aimed at optimising goal-setting skills, identifying measurable and attainable objectives, anticipating potential obstacles to these goals, and generating and sustaining the mental and physical energy required to achieve them successfully [65,66]. Hope-based interventions were found to be highly beneficial for older adults, decreasing loneliness, anxiety, hopelessness, depression, and unproductive habits in approaching problems, at the same time increasing hope, social engagement and mental health [21,67].

Laranjeira & Querido [6] summarized some evidence-based practices applicable for promoting hope: identifying and exploring one’s strengths; turning to friends and loved ones; reframing negative thoughts; engaging in pleasant activities and enhancing self-talk with positive thoughts; increasing self-esteem and self-awareness; associating with confident and optimistic people to foster “positive emotional contagion” practicing self-gratification; strengthening positive affect, training in resilience, and giving meaningful purpose to one’s life.

The results of our study clarify which components of hope specifically promote resilience in different conditions of normal old age, distinguishing them from those that are more beneficial for adults. The goal is to utilise hope as a means to counteract fear and anxiety induced by stressful events, prevent mental health issues associated with hopelessness, and enhance the well-being of individuals and social communities.

## Figures and Tables

**Table 1:** Differences between age groups in the dimensions analysed.

	Adults (50–65 Years) N = 52		Elderly (>66 Years) N = 48		Mean Dif.	Cohen’s d	t	*p*
	M	SD	M	SD				
1. CHS Spirituality	2.321	1.070	2.453	1.009	−0.133	−0.127	−0.636	0.525
2. CHS Relation/social support	3.191	0.804	3.144	0.716	0.047	0.061	0.305	0.761
3. CHS Trust	3.962	0.684	3.677	0.877	0.284	0.364	1.817	0.072
4. CHS Self-realization	3.160	0.757	2.889	0.696	0.271	0.372	1.861	0.066
5. WBP	6.867	1.362	6.182	1.345	0.685	0.506	2.527	*0* *.013*
6. RSA Social support	3.862	0.727	3.597	0.861	−0.265	−0.334	−1.667	0.099
7. RSA Future perception	3.712	0.813	3.193	0.961	−0.519	−0.585	−2.923	*0* *.004*
8. RSA Family cohesion	3.858	0.816	3.346	0.807	−0.512	−0.631	−3.151	*0* *.002*
9. RSA Personal competence	3.849	0.700	3.431	0.898	−0.419	−0.522	−2.611	*0* *.010*
10. RSA Social competence	3.685	0.784	3.396	0.870	−0.289	−0.350	−1.747	0.084
11. RSA Personal structure	2.244	0.968	2.271	0.841	0.027	0.030	0.150	0.881
12. Subjective distress	0.211	0.120	0.210	0.111	0.001	0.007	0.037	0.971

Note: all df value are 98.0; significant differences are in italics.

**Table 2:** Differences by school level in the dimensions analysed.

	Lower School N = 24		High School N = 33		University Degree N = 43		F	*p*
	M	SD	M	SD	M	SD		
1. CHS Spirituality	2.531	0.708	2.394	1.161	2.295	1.104	0.398	0.673
2. CHS Relation/social support	2.833	0.753	3.226	0.699	3.311	0.768	3.335	*0* *.040*
3. CHS Trust	3.630	0.896	3.894	0.710	3.881	0.789	0.959	0.387
4. CHS Self-realization	2.618	0.568	3.091	0.651	3.213	0.805	5.671	*0* *.005*
5. WBP	5.691	1.461	6.827	1.420	6.789	1.144	6.525	*0* *.002*
6. RSA Social support	2.535	0.921	2.162	0.857	2.194	0.659	1.835	0.165
7. RSA Future perception	2.729	0.992	2.462	0.859	2488	0.931	0.689	0.505
8. RSA Family cohesion	2.717	0.958	2.254	0.839	2.307	0.756	2.483	0.089
9. RSA Personal competence	2.389	0.806	2.232	0.796	2.422	0.865	0.523	0.594
10. RSA Social competence	2.742	0.851	2.364	0.887	2.3628	0.764	1.911	0.154
11. RSA Personal structure	3.431	0.752	3.636	1.068	4.000	0.787	3.570	*0* *.032*
12. Subjective distress	0.260	0.085	0.217	0.120	0.179	0.119	4.080	*0* *.020*

Note: significant differences are in italics.

**Table 3:** Correlations between the dimensions analysed.

	1	2	3	4	5	6	7	8	9	10	11	12	13
1. CHS Spirituality	1												
2. CHS Relation/social support	0.157	1											
3. CHS Trust	−0.198 *	0.182	1										
4. CHS Self-realization	0.131	0.585 ***	0.258 **	1									
5. WBP	0.068	0.397 ***	0.541 ***	0.547 ***	1								
6. RSA Social support	0.014	0.643 ***	0.394 ***	0.306 **	0.491 ***	1							
7. RSA Future perception	0.002	0.388 ***	0.555 ***	0.542 ***	0.656 ***	0.446 ***	1						
8. RSA Family cohesion	0.046	0.522 ***	0.468 ***	0.385 ***	0.566 ***	0.687 ***	0.487 ***	1					
9. RSA Personal competence	−0.012	0.326 ***	0.536 ***	0.481 ***	0.642 ***	0.503 ***	0.675 ***	0.547 ***	1				
10. RSA Social competence	0.051	0.348 ***	0.518 ***	0.324 **	0.568 ***	0.526 ***	0.458 ***	0.532 ***	0.587 ***	1			
11. RSA Personal structure	0.023	−0.267 **	−0.074	−0.284 **	−0.251 *	−0.313 **	−0.233 *	−0.228 *	−0.200 *	−0.138	1		
12. Subjective distress	0.092	−0.083	−0.372 ***	−0.266 **	−0.281 **	0.015	−0.204 *	−0.124	−0.111	−0.195	0.136	1	
13. Age	0.147	−0.180	−0.189	−0.321 **	−0.383 ***	−0.187	−0.290 **	−0.375 ***	−0.260 **	−0.214 *	0.063	0.124	1
M	2.384	3.168	3.825	3.030	6.538	2.265	2.538	2.388	2.352	2.454	3.743	0.211	66.08
SD	1.038	0.759	0.791	0.738	1.391	0.801	0.920	0.848	0.825	0.835	0.905	0.115	8.485
Skewness	0.318	−0.260	−0.474	−0.060	−0.700	0.357	0.478	0.195	0.539	0.085	−0.369	0.041	0.496
Kurtosis	−0.815	0.341	−0.793	−0.394	−0.033	−0.802	−0.158	−0.728	0.131	−0.733	−0.491	−0.791	−0.252
Shapiro-Wilk W	0.947	0.988	0.946	0.987	0.956	0.958	0.966	0.968	0.969	0.967	0.946	0.970	0.968
Shapiro–Wilk *p*	<0.001	0.529	<0.001	0.465	0.002	0.003	0.011	0.015	0.019	0.014	<0.001	0.022	0.014

Note. *** *p* < 0.001; ** *p* < 0.01; * *p* < 0.05.

**Table 4:** Indirect effects of the mediational model of adults.

Paths	Indirect Effect	C.I. (95%)	*p*
Subjective distress–trust–well-being	−0.18	−13.90; 0.11	0.023
Subjective distress–trust–social support	0.22	0.62; 2.99	0.004
Subjective distress–trust–future perception	0.26	0.63; 2.34	0.002
Subjective distress–trust–family cohesion	0.24	0.59; 3.07	0.006
Subjective distress–trust–personal competence	0.26	0.65; 3.51	0.005
Subjective distress–trust–social competence	0.24	0.30; 2.87	0.010

**Table 5:** Indirect effects of the mediational model of the elderly.

Paths	Indirect Effect	C.I. (95%)	*p*
Subjective distress–self-realisation–well-being	0.14	10.13; 0.91	0.031
Subjective distress–self-realisation–personal competence	0.17	0.34; 3.45	0.029
Subjective distress–relationship/social support–social support	0.27	0.69; 4.85	0.007
Subjective distress–relationship/social support–family cohesion	0.19	0.28; 3.09	0.017

## Data Availability

Data supporting this study are available on OSF at the following link: https://osf.io/vydz3/overview?view_only=3ff07c1d8ec64014b2004e3106e24759.
